# Bottom-up attention capture with distractor and target singletons defined in the same (color) dimension is not a matter of feature uncertainty

**DOI:** 10.3758/s13414-018-1538-3

**Published:** 2018-05-18

**Authors:** Hanna Weichselbaum, Ulrich Ansorge

**Affiliations:** Faculty of Psychology, University of Vienna, Liebiggasse 5, 1010 Vienna, Austria

**Keywords:** Visual search, Attention capture, Dimension weighting, Color search

## Abstract

In visual search, attention capture by an irrelevant color-singleton distractor in another feature dimension than the target is dependent on whether or not the distractor changes its feature: Capture is present if the irrelevant color distractor can take on different features across trials, but absent if the distractor takes on only one feature throughout all trials. This influence could be due to down-weighting of the entire color map. Here we tested whether a similar effect could also be brought about by down-weighting of specific color channels within the same maps. We investigated whether a similar dependence of capture on color certainty might hold true if the distractor were defined in the same (color) dimension as the target. At odds with this possibility, in the first and third blocks—in which feature uncertainty was absent—an irrelevant distractor of a certain color captured attention. In addition, in a second block, varying the distractor color created feature uncertainty, but this did not increase capture. Repeating the exact same procedure with the same participants after one week confirmed the stability of the results. The present study showed that a color distractor presented in the same (color) dimension as the target captures attention independent of feature uncertainty. Thus, the down-weighting of single irrelevant color channels within the same feature map used for target search is not a matter of feature uncertainty.

Visual attention helps humans select relevant information to fulfill their goals (Johnston & Dark, 1986). More precisely, attention is guided by (implicit or explicit) knowledge concerning a relevant (e.g., searched-for) object’s characteristic (such as its color, orientation, or size), allowing in-depth processing of the relevant object. However, even irrelevant distractor stimuli can capture attention. When irrelevant distractors are sufficiently salient, they seemingly capture attention in a *stimulus-driven* (or bottom-up) way, independent of the current search goals (cf. Itti, Koch, & Niebur, 1998; Theeuwes, 1991, 1992). For example, an irrelevant red distractor as a color singleton among several green nonsingletons seemingly does capture attention, although its color does not resemble that of the searched-for target (Theeuwes, 1991).

However, it is not entirely clear when and to what extent stimulus-driven attention capture comes to pass. In a recent study, Kerzel and Barras (2016) found that, when searching for a specific target feature, irrelevant salient distractors only captured attention when there was uncertainty about their features: During search for a shape-defined target, an irrelevant color distractor only captured attention when it changed its color randomly over the course of trials. In contrast, when the distractor had one fixed color over the course of the experiment, attention capture by the distractor was absent (see also Gaspelin & Luck, 2018). Kerzel and Barras proposed that participants down-weighted the irrelevant perceptual dimension (here, the color map) when there was certainty about the distractor color. However, when there was uncertainty about the color, down-weighting of the irrelevant color map was not possible or reasonable: “From an ecological point of view, it makes sense to ensure monitoring of new or variable features because they may correspond to potentially important changes in the environment” (Kerzel & Barras, 2016, p. 654; for related evidence, see also Gaspelin, Leonard, & Luck, 2015; Gaspelin & Luck, 2018; Theeuwes & Burger, 1998; Vatterott & Vecera, 2012).

In the present study, we wanted to test whether a similar certainty dependence exists if the irrelevant distractor and the target are defined according to the same dimension of color. Following the *dimension-weighting account* (DWA; Müller, Geyer, Zehetleitner, & Krummenacher, 2009; Müller, Reimann, & Krummenacher, 2003; Sauter, Liesefeld, Zehetleitner, & Müller, 2018)—according to which the perceptual dimension by which the target is defined is activated or “assigned weight” as a whole (cf. Treisman, 1988)—even under conditions of feature certainty, we would expect to find interference from a distractor defined by the same dimension as the target (color), because it should not be possible to down-weight the color map when the to-be found target is also defined by color. Take, for instance, the dimension of orientation, for which Liesefeld, Liesefeld, Töllner, and Müller (2017) showed attention capture by an unchanging distractor defined in the same (orientation) dimension as the target using electroencephalographic measurements (see also Sauter et al., 2018). However, there is mounting evidence that singleton suppression is feature-specific (Gaspelin & Luck, 2018; Vatterott & Vecera, 2012), and, more generally, that up-weighting of relevant features is definitely feature-specific (e.g., Folk & Remington, 1998; Wolfe, 2007). Thus, even when the defining feature of the target is realized in the same feature dimension as the irrelevant singleton distractor, it could be that successful suppression or ignorance of the irrelevant singleton distractor could be accomplished, at least if there was certainty about the distractor feature(s) and sufficient experience with these distractors (Exp. 4 of Gaspelin & Luck, 2018; Vatterott & Vecera, 2012). This could be achieved by the down-weighting of single feature channels within the same feature map that is used to find the target.

To date, only few studies have addressed this question of intradimensional bottom-up capture concerning color. Kumada (1999) and Carlei and Kerzel (2018) presented an irrelevant color-singleton distractor away from the color target during color search. Importantly, the distractor color was certain, but both studies nonetheless demonstrated interference by the color-singleton distractor during color search. However, both studies only measured manual response times (RTs). Therefore, it cannot be ruled out that the distractor interference reflected nonspatial filtering costs rather than attention capture by the distractor (Folk & Remington, 1998). Nonspatial filtering costs are RT costs due to feature (e.g., color) heterogeneity in the display that slows down the decision about where to move spatial attention.

More to the point of the present study is an experiment by Gaspar and McDonald (2014). These authors found at least some evidence that spatial capture was involved: Interference by the color-singleton distractor was stronger when the distractor was close to the target than when it was farther away from the target.^1^ However, this result was also not without complications, since interference from attention capture by singleton distractors more typically increases rather than decreases with an increasing distractor–target distance (Ansorge & Horstmann, 2007).

In the present study, we therefore compared capture by an irrelevant color distractor during color search under conditions of either certainty or uncertainty about the distractor color, and we measured eye movements to ensure that any RT interference was based on oculomotor capture and not on nonspatial filtering costs alone. There is a tight coupling between eye movements and attention (e.g., Deubel & Schneider, 1996). If irrelevant color distractors of a known certain color can capture attention during search for color targets, participants should more frequently fixate the irrelevant distractors than any nonsingleton distractor.

In detail, in the present study the distractor and target were of identical shape, differing only in their color. Participants searched for the target by its fixed known color among six disc-shaped stimuli of a different color and pressed a button in response to the stimulus inside the target (see Fig. 1 for an example of a trial).

**Fig. 1:**
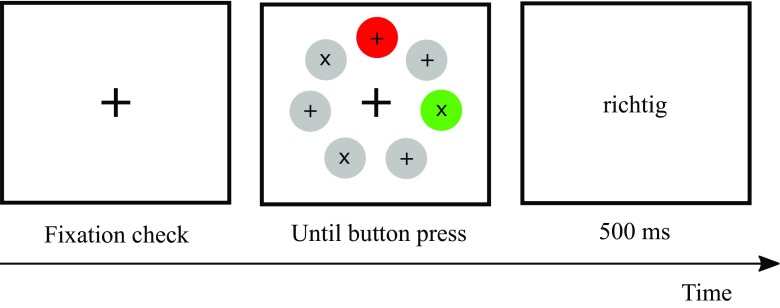
Example of a trial. The top color disc represents the target, and the color disc on the right represents an irrelevant distractor. The gray discs represent gray nonsingleton discs. The reader should please refer to the text for more information about the different specific stimulus colors. The stimuli are not drawn to scale.

In Blocks 1 and 3, participants were certain about the color of the irrelevant singleton distractors. In half of the trials of these blocks, one of the nontargets was an irrelevant singleton distractor of a known color, and this color was the same throughout these blocks. In the other half of the trials of Blocks 1 and 3, the *target-only* trials, there was no singleton distractor. If participants can suppress interference by a distractor of a known color and defined in the same dimension as the target, we expected to find no difference in the mean RTs between target-only and distractor trials. If, however, it is not possible to suppress capture by an irrelevant singleton defined along the same dimension as the target (e.g., because suppression is brought about by down-weighting of feature maps; cf. Kerzel & Barras, 2016; Müller et al., 2003), RTs should be faster in target-only than in singleton-distractor trials. Moreover, if the effect is due to attention capture, there should be more fixations on the irrelevant singleton distractors than on the nonsingletons.

Because it was possible that no capture might be observed in the feature-certain blocks, we ran a control condition in Block 2, in which participants were uncertain about the exact color of the singleton distractor. We achieved this by incorporating two differently colored distractors. Again, in one third of the trials we presented the target alone. In the remaining two thirds of trials, we equally likely presented the same irrelevant distractor as in the first block or a distractor of a different color, in this case of a color more similar to that of the target. Because the latter distractor was more similar to the target, it matched the participants’ top-down search set for the target and was, therefore, expected to lead to a stronger capture effect than the irrelevant singleton distractor (Folk, Remington, & Johnston, 1992).^2^ Since the two different singleton distractors were realized in a pseudo-random sequence, at least in Block 2, there was uncertainty regarding the singleton distractor color, and we therefore expected to find capture, and hence interference, by both the matching and the nonmatching distractor (cf. Becker, Ansorge, & Horstmann, 2009; Kerzel & Barras, 2016).

In addition, since learning could play a role in the successful suppression of the irrelevant singleton distractor (cf. Exp. 4 of Gaspelin & Luck, 2018; Vatterott & Vecera, 2012), we repeated the exact same procedure for each participant exactly one week later, on the same weekday and at the same time of day. Maybe participants could better ignore a singleton distractor under conditions of certainty during the second measurement time point. Here we also measured the individual temporal stability of the capture effect through a correlation between the two sessions. Such a correlation can help in deciding whether a numerically stable capture effect across time is also due to individually stable capture effects, in which case there should be a significant correlation, or whether numerically similar capture effects in Sessions 1 and 2 reflect mixtures of individually varying decreasing and increasing capture effects, in which case there should be no correlation.

## Method

### Participants

We tested 72 participants (*M*_age_ = 24 years, range: 19–31 years), who were mostly students and participated on a voluntary basis in return for course credit or a small monetary reward. The sample size was based on an estimation by G*Power (Faul, Erdfelder, Buchner, & Lang, 2009): Because we wanted to calculate correlations between the two recording sessions, we estimated the sample size required to observe correlation, using a bivariate normal model, two-tailed, with the parameters *α* = .05 and power = .90, assuming a correlation of *r* = .4 (cf. Versace, Mazzetti, & Codispoti, 2008). This led to a necessary sample size of 61 participants. In expectation of some dropouts, especially because the participants had to appear twice at the lab, we planned to test some extra participants, and therefore ended up testing 72. The participants had normal or corrected-to-normal vision and no dyschromatopsia, as assessed by the Ishihara color plates. They filled out a consent form prior to the experiment and were informed that data collection was fully anonymous, that the data were to be used for a scientific publication, and that they could withdraw at any time during the experiment without any consequences for them. We carefully monitored the participants’ well-being during the experiment but did not observe any inconvenience. For each participant, the experiment took about 45 min at each time point.

### Apparatus and software

The experiment was programmed using the Experiment Builder software (SR Research Ltd., Canada), and the stimuli were presented on a 19-in. CRT monitor with a resolution of 1,024 × 768 pixels. Eye movements were recorded from the dominant eye using an EyeLink 1000 Desktop Mount eyetracker (SR Research Ltd., Canada), with a sampling rate of 1,000 Hz, a gaze position accuracy of <0.5°, and a spatial resolution of <0.01°. In front of the participant, a keyboard and a standard USB computer mouse were placed. A table lamp behind the monitor served as an indirect light source. A chin rest and forehead strip ensured a viewing distance of 64 cm. The data were analyzed in the R programming environment (version 3.2.4 revised; R Core Team, 2016) using the following packages: data.table (Dowle, Short, Lianoglou, & Srinivasan, 2015), ggplot2 (Wickham, 2009), ez (Lawrence, 2015), schoRsch (Pfister & Janczyk, 2015), reshape2 (Wickham, 2007), extrafont (Winston, 2014), and Rmisc (Hope, 2013).

### Stimuli and procedure

Before the start of the experiment, we calibrated the eyetracker for each participant. In addition, to ensure appropriate calibration throughout the experiment, we executed a fixation check at the start of each trial. If, during the fixation check, the eye did not fixate inside an imaginary square with a side length of 1.3° around the fixation cross for at least 100 ms, we repeated the calibration procedure. Each trial started with the presentation of a black fixation cross in the middle of the screen. After a successful fixation check, the target display was presented. It consisted of seven discs (each 1.7° diameter) placed equidistantly on the outline of an imaginary circle with a diameter of 7.1°, starting at the 12 o’clock position. The distance between two adjacent stimuli was 3.1° from center to center (cf. Becker et al., 2009). One of these discs represented the target; the other six were nontarget discs. In each disc, a black “x” or a “+” sign was presented (0.2° × 0.2°, Arial, 10 point). In each trial, these crosses were distributed pseudorandomly across stimulus locations. Half of the trials had “x” targets and half had “+” targets, and the nontarget discs contained equal numbers of each type of cross in each trial. Participants got the instruction to first fixate the central fixation cross at the start of each trial, then to look for the target disc, which would have a specific color, and to press a button in response to the sign inside the target. Balanced across participants, they had to press one mouse button, left or right, for an “x,” and the other button for a “+.” Participants were not instructed to execute eye movements, but they were free to do so, and due to the small sizes of the crosses, eye movements were often (though not always) executed (Becker et al., 2009). After the participant’s response to the target display, 500-ms feedback at screen center indicated whether the response had been correct (“richtig” in German) or incorrect (“falsch” in German). After this feedback, the next trial started. Figure 1 depicts an example of a trial.

The experiment consisted of three consecutive blocks in which all stimuli were presented on a light gray background (LAB color coordinates 88.7/11.9/−44.5). Block 1 consisted of 168 trials, with two different trial types of equal frequency presented in pseudo-random order. *Color-singleton target trials* consisted of seven discs matched for their luminance: six gray nontarget discs (62.0 / 12.7 / − 35.8) and one target disc of one predefined color: Red 1 (62.7 / 79.0 / 65.7), Red 2 (62.0 / 76.2 / 21.1), Green 1 (62.5 / − 69.4 / 52.5), or Green 2 (62.3 / − 15.8 / 52.7). For each participant, the target was only one of these colors throughout the experiment (with target color balanced across participants). The color differences (ΔE) between Red 1 and Red 2 as well as between Green 1 and Green 2 were smaller than the other color differences. *Irrelevant distractor trials* included the same predefined color target and five gray nontarget discs, plus one green disc (if the target was red) or one red disk (if the target was green), representing an irrelevant singleton distractor. The target and irrelevant singleton distractor were always presented at alternative locations, and the positions of the target and singleton distractor were pseudo-randomly selected on each trial and counterbalanced across trials.

Block 2 was similar to Block 1, except for the additional inclusion of *target-similar singleton distractor trials*. Target-similar distractor trials consisted of one target disc and five gray discs, plus one disc having a color similar to that of the target (the Red 1 singleton distractor being similar to the Red 2 target, the Green 1 singleton distractor being similar to the Green 2 target, and vice versa) representing the target-similar singleton distractor. Block 2 consisted of 252 trials, with the same number of trials for each of its three conditions, presented in a pseudo-random order. Prior to Block 2, participants were informed that differently colored distractors would appear but that their task remained the same. Block 3 was the same as Block 1.

The experiment started with 20 practice trials, consisting of color-singleton target trials and irrelevant singleton distractor trials presented in random order that were not analyzed further. The practice trials were therefore similar to the trials of Block 1, so there were no transfer effects from a color-uncertain condition on the performance in Block 1. After practice, the 588 experimental trials (168 trials in Block 1, 252 trials in Block 2, and 168 trials in Block 3) were presented. There was a break after every 84 trials. The same experimental procedure was repeated for each participant exactly one week later, on the same day of the week and at the same time of day.

## Results

### The difference between irrelevant singleton distractor and color-singleton target trials

To analyze capture by the irrelevant singleton distractor, the manual RTs and the target fixation latencies were analyzed using a repeated measures analysis of variance (ANOVA), with the within-participant variables block (Block 1, Block 2, Block 3), condition (irrelevant singleton distractor, color-singleton target condition), and recording session (Time Point 1, Time Point 2). Note that all post-hoc *t* tests are Bonferroni-corrected.

#### Manual response times

**Fig. 2:**
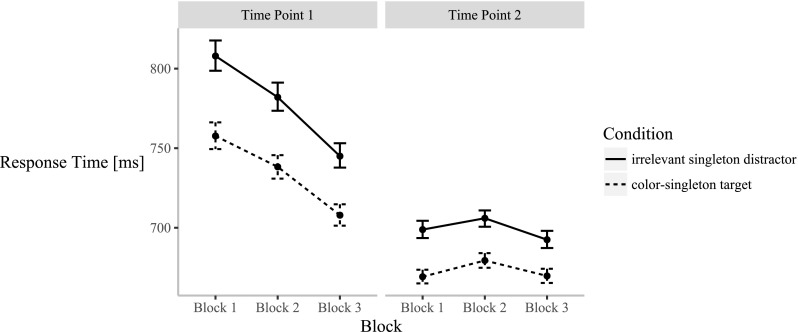
Mean response times for the irrelevant singleton distractor and color-singleton target condition, shown separately for Blocks 1–3 and Time Points 1 and 2 (error bars represent 95% confidence intervals). Note that the difference between the two conditions is significant in all blocks.

Error trials were excluded (2.90% of all trials). In line with capture by the irrelevant singleton distractor, the ANOVA of the mean RTs showed a significant effect of condition, *F*(1, 71) = 34.74, *p* < .001, ${\eta}_{\mathrm{p}}^2$ = .33, with shorter RTs in the color-singleton target condition (704 ms, 95% confidence interval (CI) [701, 706]) than in the irrelevant singleton distractor condition (739 ms, 95% CI [736, 742]). We observed a significant effect of block, *F*(2, 142) = 8.35, *p* = .002, ${\eta}_{\mathrm{p}}^2$ = .11. Reflective of some learning of the task, post-hoc *t* tests showed a significant difference between Block 1 (733 ms, 95% CI [730, 737]) and Block 3 (704 ms, 95% CI [701, 707]), *t*(71) = 3.00, *p* = .011, *d* = 0.50, as well as between Block 2 (726 ms, 95% CI [723, 730]) and Block 3, *t*(71) = 4.80, *p* < .001, *d* = 0.80, but no significant difference between Blocks 1 and 2, *t*(71) = 0.93, *p* = 1.000, *d* = 0.15. Learning or practice was also reflected in the significant effect of recording session, *F*(1, 71) = 16.43, *p* < .001, ${\eta}_{\mathrm{p}}^2$ = .19, with a shorter RT at Time Point 2 (686 ms, 95% CI [684, 688]) than at Time Point 1 (756 ms, 95% CI [753, 760]). Some learning of suppression was evident in a significant interaction between condition and block, *F*(2, 142) = 3.57, *p* = .031, ${\eta}_{\mathrm{p}}^2$ = .05. Numerically, the capture effect (difference between the irrelevant singleton distractor and the color-singleton target condition) was smaller in later than earlier blocks, but post-hoc *t* tests confirmed a significant difference between the conditions for all three blocks, Block 1: *t*(71) = 6.12, *p* < .001, *d* = 1.02; Block 2: *t*(71) = 5.76, *p* < .001, *d* = 0.96; Block 3: *t*(71) = 4.75, *p* < .001, *d* = 0.79. To check these critical effects, we additionally calculated the (scaled JZS) Bayes factor (Rouder, Speckman, Sun, Morey, & Iverson, 2009; BF_10_ shows the relative evidence for the alternative as compared to the null hypothesis). The results were strongly in favor of a difference: BF_10_ = 277,614 when comparing the conditions in Block 1, BF_10_ = 69,161 for Block 2, and BF_10_ = 1,697 for Block 3. There was no significant difference in the sizes of the capture effects between blocks: *t*(71) = 1.21, *p* = .692, *d* = 0.20, BF_01_ (= the relative evidence for the null as compared to the alternative hypothesis) = 3.84 when comparing the capture effect between Blocks 1 and 2, *t*(71) = 2.35, *p* = .064, *d* = 0.39, BF_01_ = 0.59, for Blocks 1 and 3, and *t*(71) = 1.66, *p* = .301, *d* = 0.28, BF_01_ = 2.09, for Blocks 2 and 3. See Fig. 2 for the mean RTs in the irrelevant singleton distractor and color-singleton target conditions of the three blocks, separately for Time Points 1 and 2.

In addition, we found a significant interaction between condition and recording session, *F*(1, 71) = 7.13, *p* = .009, ${\eta}_{\mathrm{p}}^2$ = .09. Again, indicative of some learning of suppression, the capture effect was numerically diminished at Time Point 2 relative to Time Point 1, but the differences between the conditions were significant at both time points, *t*(71) = 6.18, *p* < .001, *d* = 1.03, for Time Point 1, and *t*(71) = 4.05, *p* < .001, *d* = 0.68, for Time Point 2. Finally, there was a significant interaction between block and recording session, *F*(2, 142) = 9.42, *p* = .001, ${\eta}_{\mathrm{p}}^2$ = .12. Post-hoc *t* tests showed that at Time Point 1 there was no significant difference between Blocks 1 (783 ms, 95% CI[776, 789]) and 2 (760 ms, 95% CI [754, 766]), *t*(71) = 1.71, *p* = .274, *d* = 0.29, but a significant difference between Blocks 1 and 3 (726 ms, 95% CI [721, 732]), *t*(71) = 3.45, *p* = .003, *d* = 0.57, and between Blocks 2 and 3, *t*(71) = 5.01, *p* < .001, *d* = 0.83. At Time Point 2, there was only a significant difference between Blocks 2 (693 ms, 95% CI [689, 696]) and 3 (681 ms, 95% CI [678, 685]), *t*(71) = 2.46, *p* = .049, *d* = 0.41, showing a significantly higher mean RT in Block 2 than in Block 3, but no significant difference between Blocks 1 (684 ms, 95% CI [681, 687]) and 2, *t*(71) = − 1.98, *p* = .154, *d* = − 0.33, and between Blocks 1 and 3, *t*(71) = 0.49, *p* = 1.00, *d* = 0.08. There was no significant three-way interaction, *p* = .656. The means of all conditions are presented in Table 2 in the Appendix 1.

#### Target fixation latencies

This analysis can be found in Appendix 2.

#### Error analysis

To exclude a possible speed–accuracy trade-off, we analyzed the arcsine-transformed error rates using the same ANOVA as for the RTs and target fixation latencies. There were neither significant main effects nor any significant interaction, all *p*s > .069.

### The difference between target-similar singleton distractor, irrelevant singleton distractor, and color-singleton target trials in Block 2

The manual RTs and the target fixation latencies were analyzed using a repeated measures ANOVA, with the within-participant variables condition (target-similar singleton distractor, irrelevant singleton distractor, color-singleton target condition) and recording session (Time Point 1, Time Point 2).

#### Manual response times

Error trials were excluded (3.22% of all trials). Reflective of stimulus-driven capture and top-down contingent capture, the ANOVA showed a significant effect of condition, *F*(2, 142) = 90.91, *p* < .001, ${\eta}_{\mathrm{p}}^2$ = .56, with significant differences between all three conditions: *t*(71) = 9.03, *p* < .001, *d* = 1.50, between the irrelevant singleton distractor condition (744 ms, 95% CI [739, 749]) and the target-similar singleton distractor condition (847 ms, 95% CI [839, 855]); *t*(71) = 10.53, *p* < .001, *d* = 1.76, between the target-similar distractor condition and the color-singleton target condition (709 ms, 95% CI [704, 713]); and *t*(71) = 5.76, *p* < .001, *d* = 0.96, between the irrelevant singleton distractor and color-singleton target conditions. Indicative of a general learning or practice effect, the effect of recording session was significant, *F*(1, 71) = 21.07, *p* < .001, ${\eta}_{\mathrm{p}}^2$ = .23, too, with shorter RTs at Time Point 2 (725 ms, 95% CI [721, 728]) than at Time Point 1 (808 ms, 95% CI [802, 815]). In addition, some learning to suppress the distractor led to a significant interaction between condition and recording session, *F*(2, 142) = 18.66, *p* < .001, ${\eta}_{\mathrm{p}}^2$ = .21. However, at Time Points 1 and 2 there were capture effects by both types of distractors, since the difference between the conditions was significant: We observed significant differences between the irrelevant singleton distractor and target-similar distractor conditions, *t*(71) = 8.24, *p* < .001, *d* = 1.37, for Time Point 1, and *t*(71) = 8.56, *p* < .001, *d* = 1.43, for Time Point 2; between the target-similar distractor and color-singleton target conditions, *t*(71) = 9.98, *p* < .001, *d* = 1.66, for Time Point 1, and *t*(71) = 9.72, *p* < .001, *d* = 1.62, for Time Point 2; and between the irrelevant singleton distractor and color-singleton target conditions, *t*(71) = 5.62, *p* < .001, *d* = 0.94, for Time Point 1, and *t*(71) = 4.23, *p* < .001, *d* = 0.71, for Time Point 2. The means of all conditions are presented in Table 3 in the Appendix 1.

#### Target fixation latencies

This analysis can be found in Appendix 2.

#### Error analysis

Analyzing the arcsine-transformed error rates revealed a significant effect of condition, reflecting top-down contingent attentional capture by the target-similar distractors, *F*(2, 142) = 5.36, *p* = .006, ${\eta}_{\mathrm{p}}^2$ = .07, with a significant difference between the target-similar singleton distractor (.17, 95% CI [.16, .19]) and the irrelevant singleton distractor (.14, 95% CI [.13, .16]), *t*(71) = 3.11, *p* = .008, *d* = 0.52. The differences between the color-singleton target condition (.15, 95% CI [.14, .17]) and both other conditions were not significant: *t*(71) = 2.24, *p* = .084, *d* = 0.37, for the target-similar distractor condition, and *t*(71) = − 0.90, *p* = 1.00, *d* = − 0.15, for the irrelevant singleton distractor condition. Learning was also observed: There was a significant effect of recording session, *F*(1, 71) = 7.00, *p* = .010, ${\eta}_{\mathrm{p}}^2$ = .09, with a lower error rate at Time Point 2 (.15, 95% CI [.14, .16]) than at Time Point 1 (.16, 95% CI [.15, .18]). The interaction was not significant, *p* = .116.

### Distractor fixation frequencies

To guarantee that the prolonged RTs were due to the spatial capture of attention (cf. Folk & Remington, 1998), we compared the mean numbers of fixations on the distractors. In line with capture, the irrelevant singleton distractors (0.13, 95% CI [0.09, 0.17]) and the target-similar singleton distractors (0.46, 95% CI [0.39, 0.53]) were both fixated more often than the gray discs (0.03, 95% CI [0.02, 0.04], for the irrelevant singleton distractor condition and the target-similar singleton distractor condition, as well), *t*(71) = − 5.29, *p <* .001, *d* = − 0.88, for the irrelevant singleton distractor condition, and *t*(71) = − 11.84, *p <* .001, *d* = − 1.97, for the target-similar singleton distractor condition. In addition, we found evidence of top-down contingent capture in the form of a significant difference in mean distractor fixations between the target-similar and irrelevant singleton distractors, *t*(71) = 10.32, *p <* .001, *d* = 1.72.

### Feature versus singleton search

Trials containing only a color-singleton target—one half of all trials in Blocks 1 and 3, and one third of all trials in Block 2—made it possible to search for a singleton irrespective of its color in order to find the target (cf. Bacon & Egeth, 1994). It is therefore possible that, on the basis of these trials, participants incorporated a top-down search setting for singletons, irrespective of their color. As a consequence, the irrelevant singleton distractor would have captured attention in a top-down-contingent way because of its singleton status. Because this would have compromised the rationale of the present experiment, we tested whether the stable capture effects by the irrelevant distractor singleton could have reflected passive carryover of a top-down singleton search setting from a preceding color-singleton target trial to a distractor trial: We tested whether interference by the irrelevant singleton distractor was boosted in trials following color-singleton target trials (in which a singleton search strategy was possible) relative to trials following a distractor trial (in which a singleton search strategy was not possible). This was not the case. On the contrary, collapsing over blocks and time points, we found a significant effect of the condition in the preceding trial, *F*(2, 142) = 8.93, *p* = .001, ${\eta}_{\mathrm{p}}^2$ = .11, on the RTs in irrelevant singleton distractor trials, but the RT of the irrelevant singleton distractor trial was *lower* when it was preceded by a color-singleton target trial (731 ms, 95% CI [727, 735], for the irrelevant singleton distractor trial) than when it was preceded by another irrelevant singleton distractor trial (741 ms, 95% CI [736, 745]), *t*(71) = 2.87, *p* = .016, *d* = 0.48, or by a target-similar singleton distractor trial (750 ms, 95% CI [741, 759]), *t*(71) = 3.80, *p* = .001, *d* = 0.63. There was no significant difference in the RTs of irrelevant singleton distractor trials when they were preceded by an irrelevant singleton distractor trial or by a target-similar singleton distractor trial, *t*(71) = 2.02, *p* = .140, *d* = 0.34. Analyzing the target fixation latencies revealed no significant effect of the preceding condition, *p* = .146.

### Correlations between Time Points 1 and 2

Now that we know that irrelevant singleton distractors led to significant capture effects throughout all blocks, but that learning diminished these effects somewhat on average, we wanted to know whether more learning of suppression was hidden in the averages because some participants may have shown increased capture effects across time. To assess such interindividual variability versus the stability of capture by the irrelevant singletons across time, we calculated correlations of the capture effects by irrelevant singleton distractors and by target-similar singleton distractors between Time Points 1 and 2. The capture effect of the irrelevant singleton distractor was calculated as the difference between the irrelevant singleton distractor condition and the color-singleton target condition. The capture effect of the target-similar singleton distractor was calculated as the difference between the target-similar singleton distractor condition and the irrelevant singleton distractor condition, since the two distractors were equally salient,^3^ and therefore the difference between the two would represent any additional capture based on the match of the target-similar singleton distractor with the top-down search setting for the targets or the stronger priming of attention capture of the target-similar singleton distractor by a target in the preceding trial. All correlations are in Table 1. Note that we report Spearman correlations, since the distributions were not normal because of a few outliers. As can be seen, all correlations were significant, meaning that an average capture effect did not underestimate suppression of capture by the (irrelevant) singleton distractors.

**Table 1:** Spearman correlations of manual response times (RTs) and target fixation latencies (TFLs) between Time Point 1 and Time Point 2

Block	Effect	Spearman’s Rho for RTs	Spearman’s Rho for TFLs
1	Irrelevant singleton distractor	.26^*^	.50^*^
2	Irrelevant singleton distractor	.31^*^	.39^*^
2	Target-similar distractor	.85^*^	.80^*^
3	Irrelevant singleton distractor	.32^*^	.48^*^

Significant correlations (*p <* .05) are marked by an asterisk.

## Discussion

In the present study, we tested capture by an irrelevant singleton distractor defined along the same feature dimension (color) as the searched-for color target, under two types of conditions: conditions with certainty and with uncertainty about the distractor color. Independently of whether there was or was not certainty about the singleton distractor color, the irrelevant singleton distractor captured attention, and the amounts of capture measured by manual RTs did not differ significantly.

According to a recent study by Kerzel and Barras (2016), when participants were certain about the color of an irrelevant singleton distractor during search for a shape target, the capture effect was eliminated. Kerzel and Barras attributed this effect to the down-weighting of the whole color map, but it could also be attributed to the down-weighting of single features of the color map. The aim of the present study was to tell these two possibilities apart. We reasoned that if down-weighting concerned single colors rather than the whole color map, we should be able to find a similar influence of uncertainty about the particular distractor color in a condition in which the target is defined by a feature in the same dimension (here: color) as the distractor. However, capture by the irrelevant singleton distractor was not significantly different in the first and third blocks, in which participants were certain about the distractor color, from the second block, in which participants were uncertain about the distractor color. This is in line with Müller et al.’s (2003) DWA, arguing that feature maps (here, for color) are down-weighted as a whole—a strategy that is not viable if the target is defined by the same dimension as the distractor. This is also in line with Kerzel and Barras’s hypothesis that distractors presented in the same dimension as the target should interfere with search. In addition, capture by the irrelevant singleton distractor did not vary much between early and late blocks or between the first and second measurement time points, ruling out strong practice effects of distractor suppression in our experiment (cf. Vatterott & Vecera, 2012).^4^ This interpretation was also supported by significant correlations between the capture effects of irrelevant singleton distractors in Sessions 1 and 2. These correlations showed that the stable capture effects by the irrelevant singleton distractors were not due to a mixture of individuals showing increasing suppression and other individuals showing the opposite trend, increasing capture effects, across time.

What is more, as we included target-similar distractors in the second block, we were also able to show that the capture effect of the irrelevant singleton distractor was not due to top-down singleton search. Top-down singleton search should have allowed for similar capture effects by target-similar and irrelevant singleton distractors. The higher capture effect of the target-similar distractors was thus indicative of feature search for target color, so that the match of the target-similar singleton distractor to a top-down search setting for target color created an additional capture effect. Now we are aware that inter-trial priming of capture by the target-similar capture through its resemblance to the color target in the preceding trial could likewise have boosted the capture effect of the target-similar singleton distractor. However, the fact that whether or not a preceding trial allowed top-down singleton search had not the expected influence on capture by the irrelevant singleton distractor speaks against an explanation in terms of top-down singleton search, too. Top-down singleton search would have been only possible in target-singleton trials, so that interference by the irrelevant singleton distractor should have been particularly strong following such a target-singleton trial. However, the opposite was found: For whatever reason, interference by the irrelevant singleton distractor was lowest following a target-singleton trial. This finding is highly suggestive that our participants were not in a top-down singleton search mode and that the capture effect of the irrelevant singleton distractor we found reflected bottom-up capture by feature salience.

Furthermore, interference in the additional-singleton protocol of Theeuwes (1991, 1992) has sometimes been attributed to a nonspatial filtering cost (cf. Folk & Remington, 1998). However, in the present study we were able to demonstrate the spatial nature of the interference effect by demonstrating that the eyes were also attracted by the irrelevant singleton distractors: Fixation frequencies were higher on irrelevant singleton distractors than on any of the nonsingleton stimuli. Because eye movements and attention shifts are tightly coupled (cf. Deubel & Schneider, 1996), the fixation frequency effect supports the interpretation of the interference effect as not only a spatial effect, but also an attentional effect.

Because we carried out the experiment twice with the same participants (as was necessary to study possible training effects), overall, the RTs got faster. However, most importantly, the capture effect by the irrelevant singleton distractor did not change much across blocks. Analyzing the error trials confirmed all the results and ruled out a possible speed–accuracy trade-off. This means that participants got better at the task at hand. Nonetheless, checking the stability of the capture effect of the irrelevant singleton distractor, we found significant correlations between Time Points 1 and 2. Admittedly, these correlations were not as high as for the capture effect by target-similar singleton distractors, but together with the significant capture effects of the irrelevant singleton distractor at both time points and in all blocks, the data speak of robust and reliable bottom-up capture effects from even known and certain irrelevant singleton distractors, and thus confirm the explanation of Kerzel and Barras (2016). For the same reason, our findings also support the conclusions of former studies that claimed a similar impossibility to entirely suppress irrelevant singletons presented in the same dimension as the relevant targets (Carlei & Kerzel, 2018; Gaspar & McDonald, 2014; Kumada, 1999). The present results thus also shed a light on past demonstrations of successful suppression of color distractors. Gaspelin and Luck (2018, Exp. 4) asked their participants to search for shape-defined targets and Vatterott and Vecera (2012) asked their participants to search for shape- and color-defined targets, and both studies showed that at least with practice an irrelevant singleton distractor of a known fixed color could be successfully suppressed. Yet, both of these studies were silent on the potential role of different maps for relevant target features versus irrelevant distractor features for successful distractor suppression. In light of the present study, it seems clear that the usage of different maps for target versus distractor features was likely responsible for the successful suppression of irrelevant color distractors in these studies.

Although in the present study we focused on the feature dimension of color, we think that similar principles will apply to other feature dimensions. For example, several studies have incorporated irrelevant distractors that were fixed and therefore certain in other feature dimensions and found evidence of attention capture once the target was realized in the same dimension. For instance, Zehetleitner, Goschy, and Müller (2012) presented a shape-defined distractor together with a shape-defined target. Although the distractor did not vary, it nonetheless caused interference. The authors reasoned that participants were unable to suppress intradimensional interference. This is in accordance with our result in the color domain. Similarly, van Zoest, Donk, and Theeuwes (2004) measured saccade latencies to an orientation-defined target line presented together with a salient right- or left-tilted distractor among vertically oriented nonsingleton lines. These authors also found capture by the distractor (see also Liesefeld et al., 2017; Sauter et al., 2018).

Note, however, that these studies did not use color and did not vary distractor certainty, as was done in the present study. Thus, it is unclear whether participants can suppress fixed, known color distractors during search for color targets and whether distractor interference in these protocols would be boosted if there were more uncertainty about the distractor features. In other words, the evidence for a failure of suppression of irrelevant distractors realized in the same dimension as the target in these studies was more indirect than in the present study, and future investigations should check whether certainty has no influence on distractor interference in these feature domains, too.

To sum up, our study showed interference by an irrelevant color-singleton distractor when participants searched for a target defined by the same color dimension. This was independent of the participants’ certainty about the distractor color. In addition, by measuring eye movements, we showed that the distractor interference was based on attention capture. Thus, it is probably difficult to suppress bottom-up attention capture by an irrelevant singleton distractor once this distractor is realized in the same feature dimension that is also used to search for the targets.

### Author note

The authors declare no conflicts of interest.

## Appendix 1

**Table 2:** Means and 95% confidence intervals (CIs) of manual response times (RTs) and target fixation latencies (TFLs) of the color-singleton target and the irrelevant singleton distractor conditions, separately for blocks and time points

Condition	Block	Recording Session	Mean RTs and CIs (in ms)	Mean TFLs and CIs (in ms)
Color-singleton target	1	Time Point 1	758 [749, 766]	225 [223, 227]
Irrelevant singleton distractor	1	Time Point 1	808 [798, 817]	261 [258, 265]
Color-singleton target	2	Time Point 1	738 [731, 746]	223 [220, 225]
Irrelevant singleton distractor	2	Time Point 1	782 [773, 791]	250 [247, 252]
Color-singleton target	3	Time Point 1	708 [701, 714]	225 [223, 228]
Irrelevant singleton distractor	3	Time Point 1	745 [737, 753]	250 [247, 253]
Color-singleton target	1	Time Point 2	669 [665, 673]	210 [208, 212]
Irrelevant singleton distractor	1	Time Point 2	699 [694, 704]	234 [231, 236]
Color-singleton target	2	Time Point 2	679 [675, 684]	218 [216, 220]
Irrelevant singleton distractor	2	Time Point 2	706 [701, 711]	240 [238, 243]
Color-singleton target	3	Time Point 2	670 [665, 674]	219 [217, 221]
Irrelevant singleton distractor	3	Time Point 2	692 [687, 698]	238 [236, 241]

**Table 3:** Means and 95% confidence intervals (CIs) of manual response times (RTs) and target fixation latencies (TFLs) of all conditions of Block 2, separately for time points

Condition	Recording Session	Mean RTs and CIs (in ms)	Mean TFLs and CIs (in ms)
Color-singleton target	Time Point 1	738 [731, 746]	223 [220, 225]
Irrelevant singleton distractor	Time Point 1	782 [773, 791]	250 [247, 252]
Target-similar distractor	Time Point 1	906 [892, 921]	316 [312, 320]
Color-singleton target	Time Point 2	679 [675, 684]	218 [216, 220]
Irrelevant singleton distractor	Time Point 2	706 [701, 711]	240 [238, 243]
Target-similar distractor	Time Point 2	789 [782, 795]	299 [296, 303]

## Appendix 2 Target fixation latencies

### Irrelevant singleton distractors versus color-singleton target trials

Trials with no target fixation were excluded (7.72% of the trials included in the RT analysis). Reflective of capture by the irrelevant singleton distractors, the ANOVA showed a significant effect of condition, *F*(1, 71) = 66.90, *p* < .001, ${\eta}_{\mathrm{p}}^2$ = .49, with a shorter latency in the color-singleton target trials (220 ms, 95% CI [219, 221]) than in the irrelevant singleton distractor trials (245 ms, 95% CI [244, 247]). Learning or practice led to a significant main effect of recording session, *F*(1, 71) = 9.54, *p* = .003, ${\eta}_{\mathrm{p}}^2$ = .12, with a shorter target fixation latency at Time Point 2 (227 ms, 95% CI [226, 227]) than at Time Point 1 (239 ms, 95% CI [238, 240]). Indicative of learning to suppress the distractors, there was a significant interaction between condition and block, *F*(2, 142) = 8.82, *p* = .001, ${\eta}_{\mathrm{p}}^2$ = .11, but, again, post-hoc *t* tests showed that the difference between the irrelevant singleton distractor condition and the color-singleton target condition was significant for all three blocks: Block 1, *t*(71) = 8.26, *p* < .001, *d* = 1.38, BF_10_ = 1,580,210,419; Block 2, *t*(71) = 7.95, *p* < .001, *d* = 1.32, BF_10_ = 441,856,056; and Block 3, *t*(71) = 6.86, *p* < .001, *d* = 1.14, BF_10_ = 5,221,427. Again, there was no significant difference in the capture effects between Blocks 1 (31 ms, 95% CI [23, 38]) and 2 (26 ms, 95% CI [19, 32]), *t*(71) = 2.12, *p* = .113, *d* = 0.35, BF_01_ = 0.94, but significant differences in the capture effects between Blocks 1 and 3 (22 ms, 95% CI [16, 28]), *t*(71) = 3.65, *p* = .001, *d* = 0.61, BF_10_ = 47.33, and between Blocks 2 and 3, *t*(71) = 2.70, *p* = .026, *d* = 0.45, BF_10_ = 3.69.

Learning to suppress the distractors also led to a significant interaction between condition and recording session, *F*(1, 71) = 10.02, *p* = .002, ${\eta}_{\mathrm{p}}^2$ = .12. We found a significant difference between the two conditions for both recording sessions—Time Point 1, *t*(71) = 8.34, *p* < .001, *d* = 1.39; Time Point 2, *t*(71) = 6.96, *p* < .001, *d* = 1.16—but the capture effect was weaker at Time Point 2. Finally, there was also a significant interaction between block and recording session, *F*(2, 142) = 9.42, *p <* .001, ${\eta}_{\mathrm{p}}^2$ = .12. The patterns of significance were very similar at both time points. At Time Point 1, no significant difference emerged between Blocks 1 and 3, *t*(71) = 1.22, *p* = .676, *d* = 0.20, or between Blocks 2 and 3, *t*(71) = − 1.04, *p* = .909, *d* = − 0.17, but a difference did emerge between Blocks 1 and 2, *t*(71) = 2.51, *p* = .043, *d* = 0.42. At Time Point 2, again, we found no significant difference between Blocks 1 and 3, *t*(71) = − 1.82, *p* = .217, *d* = − 0.30, or between Blocks 2 and 3, *t*(71) = 0.07, *p* = 1.00, *d* = 0.01, but there was one between Blocks 1 and 2, *t*(71) = − 3.28, *p* = .005, *d* = − 0.55. Note however, that at Time Point 2 the target fixation latency was shorter in Block 1 (222 ms, 95% CI [220, 223]) than in Blocks 2 (229 ms, 95% CI [227, 231]) and 3 (229 ms, 95% CI [227, 230]), whereas at Time Point 1, this latency was longer in Block 1 (243 ms, 95% CI [241, 245]) than in Blocks 2 (236 ms, 95% CI [234, 238]) and 3 (238 ms, 95% CI [236, 240]). Thus, participants started from a much faster search-time level in Session 2, leaving little room for further general speedup of their responses. We observed no significant effect of block and no significant three-way interaction, both *p*s > .155. The means of all conditions are presented in Table 2 in Appendix 1.

### Performance in Block 2: Irrelevant singleton distractors versus target-similar distractors versus color-singleton target trials

Trials with no target fixations were excluded (8.02% of the trials included in the RT analysis). We did find capture effects: The ANOVA showed a significant effect of condition, *F*(2, 142) = 160.81, *p* < .001, ${\eta}_{\mathrm{p}}^2$ = .69, with significant differences between all three conditions: *t*(71) = 11.14, *p* < .001, *d* = 1.86, between the irrelevant singleton distractor condition (245 ms, 95% CI [243, 247]) and the target-similar singleton distractor condition (308 ms, 95% CI [305, 311]); *t*(71) = 15.38, *p* < .001, *d* = 2.56, between the target-similar distractor condition and the color-singleton target condition (220 ms, 95% CI [219, 222]); and *t*(71) = 7.95, *p* < .001, *d* = 1.32, between the irrelevant singleton distractor and the color-singleton target condition. Learning was also demonstrated, in that the effect of recording session was nearly significant, *F*(1, 71) = 3.98, *p* = .050, ${\eta}_{\mathrm{p}}^2$ = .05, with a shorter latency at Time Point 2 (252 ms, 95% CI [251, 254]) than at Time Point 1 (263 ms, 95% CI [261, 264]). Learning to suppress the distractor was reflected in a significant interaction between condition and recording session, *F*(2, 142) = 6.16, *p* = .005, ${\eta}_{\mathrm{p}}^2$ = .08. However, again, capture was never entirely eliminated: At Time Points 1 and 2, the difference between the conditions was significant. There were significant differences between the irrelevant singleton distractor and the target-similar distractor conditions, *t*(71) = 10.91, *p* < .001, *d* = 1.82, for Time Point 1, and *t*(71) = 9.51, *p* < .001, *d* = 1.59, for Time Point 2; between the target-similar distractor and the color-singleton target conditions, *t*(71) = 14.51, *p* < .001, *d* = 2.42, for Time Point 1, and *t*(71) = 14.59, *p* < .001, *d* = 2.43, for Time Point 2; and between the irrelevant singleton distractor and color-singleton target conditions, *t*(71) = 7.81, *p* < .001, *d* = 1.30, for Time Point 1, and *t*(71) = 6.83, *p* < .001, *d* = 1.14, for Time Point 2. The means of all conditions are presented in Table 3 in Appendix 1.

## Footnotes

1 Gaspar and McDonald (2014) also used lateralized event-related potentials to test whether irrelevant distractors captured attention. Interestingly, however, their results were equivocal with respect to the possibility of attention capture by the irrelevant singleton distractors, since they only found evidence of active suppression of the irrelevant singleton distractor positions (or sides), and the authors themselves interpreted their findings as suppression, but not as attention capture.

2 Note that prior research had shown that target similarity of the singleton distractor is sufficient for top-down-dependent capture effects (Anderson & Folk, 2010; Ansorge & Heumann, 2003, 2004).

3 Saliency is calculated as a local color difference (Itti et al., 1998). Therefore, target-similar distractors could have been slightly less salient than non-matching distractors if they were presented directly adjacent to the target. However, targets and distractors next to each other were realized in only 2/6 of the target-similar distractor trials. Therefore, we neglected this potential difference.

4 Note that in our experiment the extent of practice with the irrelevant singleton was altogether 504 trials, and that this compares to practice with each irrelevant singleton in 24 trials in Vatterott and Vecera (2012) and 60 trials in Experiment 4 of Gaspelin and Luck (2018). This calculation is based on the block lengths of 48 and 120 trials in Vatterott and Vecera and in Gaspelin and Luck’s Experiment 4, respectively, each of which contained a new to-be-learned irrelevant singleton and 50% no-singleton distractor trials. Thus, a lack of practice in our experiment cannot account for our results.
